# Dynamical system analysis of FLRW models with Modified Chaplygin gas

**DOI:** 10.1038/s41598-020-80396-w

**Published:** 2021-02-02

**Authors:** Ali Osman Yılmaz, Ertan Güdekli

**Affiliations:** Department of Physics, Istanbul University, Istanbul, 34134 Turkey

**Keywords:** Cosmology, Dark energy and dark matter, Early universe, General relativity and gravity

## Abstract

We investigate Friedmann–Lamaitre–Robertson–Walker (FLRW) models with modified Chaplygin gas and cosmological constant, using dynamical system methods. We assume $p=(\gamma -1)\mu -\dfrac{A}{\mu ^\alpha }$ as equation of state where $\mu$ is the matter-energy density, *p* is the pressure, $\alpha$ is a parameter which can take on values $0<\alpha \le 1$ as well as *A* and $\gamma$ are positive constants. We draw the state spaces and analyze the nature of the singularity at the beginning, as well as the fate of the universe in the far future. In particular, we address the question whether there is a solution which is stable for all the cases.

## Introduction

This study is about the evolution of spatially homogeneous cosmological models which includes a cosmological constant. First of all, it is extremely difficult and even impossible to find the solution of a system consisting of nonlinear, coupled n partial differential equations. The only thing to do is to use either perturbation or numerical solution methods. However, there is another method that allows some critical properties to be determined about the solutions without knowing the exact solution of the system of equations. In such an approach to a system of differential equations; a dynamical system is introduced by making the variables dimensionless as well as redefining the differentiation variable to cover all of the IR, and then dynamical system theory (DST), which is introduced by Poincare at the end of the nineteenth century, is applied^1–3^. Although it has improved a lot, DST is still a raw theory. Its deficiencies constitute an intensive research area today. The method called qualitative analysis lead many scientist to work on this subject^3,4^.

Since the field equations of the general relativity theory are of the type mentioned above, they form a challenging system of equations. Even though it is possible to reduce the number of equations or variables by assuming properties about spacetime such that spherical symmetry, homogeneity and isotropy, it is still not easy to find a solution. This is also the case in the field of cosmology. Although the assumptions of homogeneity and isotropy, which are extremely restrictive for the spacetime, lead the cosmological evolution equations to become second-order and linear, analytical solutions are handled in Friedmann–Lemaître–Robertson–Walker (FLRW) models only for some specific cases^5,6^.

DST was applied to constraint and evolution equations first by Collins in 1971^7^. Actually, DST responds precisely to the request in cosmology. As already known, many observational data, such as counting of galaxies and cosmic microwave background radiation measurements lead to the assumption that the universe is homogeneous and isotropic. This provides a basis for the widespread use of FLRW models. So the description of the following two universes is of great importance for the evolution of the universe: the nature of the singularity at the beginning, and the fate of the universe in the far future.

There are many studies in the literature about the applications of DST in cosmology^8–14^. The common approach is to assume a perfect-fluid matter source with $p=(\gamma -1)\mu$ as equation of state but recently Chaplygin gas with $p=-\dfrac{A}{\mu }$ as equation of state was introduced as a candidate for unified model of dark energy^15^. Later this was generalized to generalized Chaplygin gas with $p=-\dfrac{A}{\mu ^\alpha }$ as equation of state where $0<\alpha \le 1$^16–18^. This equation of state was later modified to $p=B\mu -\dfrac{A}{\mu ^\alpha }$ where $B>0$ and $0<\alpha \le 1$^19,20^. This one is called Modified Chaplygin Gas.

Copeland et al.^21^ reviewed in detail some approaches which are used to explain the acceleration of the universe and presented some dark energy models. Bahamonde et al.^22^ studied the applications of dynamical system to cosmology, focusing on the accelerated expansion of the universe. Jing et al.^23^ studied the dynamical attractor of the modified Chaplygin gas model and found that there are no interaction between barotropic background fluid and modified Chaplygin gas as well as modified Chaplygin gas will behave similar to $\Lambda$CDM in the far future. Li et al.^24^ studied the modified Chaplygin gas model that interacts with cold dark matter. Bhadra and Debnath^25^ studied an interacting modified Chaplygin gas model, using dynamical system methods. Fang et al.^26^ considered a potential parameter $\Gamma (\lambda )$ and made a three dimensional analysis of quintessence scalar field.

In this study, we use modified Chaplygin gas with $p=(\gamma -1)\mu -\dfrac{A}{\mu ^\alpha }$ as equation of state where $\gamma$ is a parameter which is in the interval $0 \le \gamma \le 2$, $\mu$ is the matter-energy density, *p* is the pressure, $\alpha$ is a parameter which can take on values $0<\alpha \le 1$ as well as *A* is a positive constant.It is clear that when $\gamma =1$, generalized Chaplygin gas is recovered. If $\alpha =1$ as well, it reduces to pure Chaplygin gas while $A=0$ recovers the equation of state of perfect fluid^15,27,28^. We also include the nonnegative cosmological constant in our study. Therefore, we obtain a three dimensional state space where a mixture of perfect fluid, generalized Chaplygin gas and cosmological constant exist.

The outline of the paper is as following. In section “Methods”, we show how the dynamical system is obtained. In “Results” section, using Friedmann equation and weak energy condition we obtain the constraints of the state spaces; find the equilibrium points where all the derivatives are zero; obtain the eigenvalues using the linearized Jacobian matrix as well as construct the three dimensional complete state space. We end with some conclusions in “Discussion” section.

## Methods

FLRW models are spatially homogeneous and isotropic models and represented by the following metric in a $(t,r,\theta ,\psi )$ co-moving coordinate system.

1 $$\begin{aligned} \mathrm {d}s^2=-\mathrm {d}t^2+l^2(t)[\mathrm {d}r^2+f_k^2 (r)(\mathrm {d}\theta ^2+sin^2 (\theta ) \mathrm {d}\psi ^2 )] \end{aligned}$$

This metric which is called Robertson-Walker (RW) metric is independent of field equations. $f_{k}$(r) function, which depends on the parameter k, is defined as following.

2 $$\begin{aligned} f_k(r)={\left\{ \begin{array}{ll} \sin {r} & k=+1 \\ r & k=0\\ \sinh {r} & k=-1 \end{array}\right. } \end{aligned}$$

According to this; universe is elliptical if $k=+1$, Euclidean if $k=0$ and hyperbolic if $k=-1$. *t* and *l*(*t*) are called cosmic time and distance scale factor, respectively.

Defining Hubble scalar by $H={\dot{l}}/l$, the field equations and equations of motion become as following^29^

3 $$\begin{aligned} H^2&= \frac{1}{3}\mu -\frac{1}{6}{}^3R+\frac{1}{3}\Lambda \end{aligned}$$

4 $$\begin{aligned} \frac{\ddot{l}}{l}&= -qH^2 = -\frac{3p+\mu }{6}+\frac{1}{3}\Lambda \end{aligned}$$

5 $$\begin{aligned} {\dot{\mu }}&= -3H(\mu +p) \end{aligned}$$

Here Eqs. (3–5) are the Friedmann equation, the Raychaudhuri equation and the energy conservation equation, respectively where ${}^3R$ is the three-curvature of the symmetry surfaces, *q* is the deceleration parameter, $\mu$ is the energy density, *p* is the pressure and $\Lambda$ is the cosmological constant as well as a dot denotes the derivative with respect to cosmic time.

We use Eq. 6 as equation of state.

6 $$\begin{aligned} p=(\gamma -1)\mu -\dfrac{A}{\mu ^\alpha } \end{aligned}$$

In order to make the variables dimensionless, it is necessary to norm them to a suitable variable. This suitable variable could be determined with the help of Eq. (3).

Matter-energy density $\mu$ is nonnegative because of the weak energy condition and ${}^3R \le 0$ if $k=0$ or $k=-1$. If $\Lambda$ is nonnegative $\left( \Lambda \ge 0\right)$ as well, the term $H^2$ becomes greater than the other terms so it becomes the dominant term.

Therefore, the variables could be made dimensionless by dividing them by the powers of *H* if $H \ne 0$ . According to this, for $k=0,-1$ and $\Lambda \ge 0$, the following dimensionless variables can be defined where $\Omega$, $\Omega _\Lambda$, *K*, $\Omega _A$ and *P* are matter density, $\Lambda$ density, curvature, Chaplygin gas density and pressure parameters, respectively which are dimensionless.

7 $$\begin{aligned} \begin{aligned} \Omega =\frac{\mu }{3H^2}, \qquad \Omega _\Lambda =\frac{\Lambda }{3H^2}, \qquad K=-\frac{{}^3R}{6H^2}, \qquad \Omega _A = \dfrac{\root \alpha + 1 \of {A}}{3H^2}, \qquad P=\frac{p}{3H^2} \equiv (\gamma -1)\Omega - \dfrac{\Omega _A^{\alpha +1}}{\Omega ^\alpha } \end{aligned} \end{aligned}$$

Considering these dimensionless variables as well as defining a dimensionless time variable as $d\tau =H(t)dt$, we obtain following evolution and constraint equations as well as the definition equation of q.

8 $$\begin{aligned} H'&= -\left( 1+q\right) H \end{aligned}$$

9 $$\begin{aligned} \Omega '&= (2q - 1)\Omega -3\left( (\gamma -1)\Omega - \dfrac{\Omega _A^{\alpha +1}}{\Omega ^\alpha }\right) \end{aligned}$$

10 $$\begin{aligned} \Omega _\Lambda '&= 2(1+q)\Omega _\Lambda \end{aligned}$$

11 $$\begin{aligned} \Omega _A'&= 2(1+q)\Omega _A \end{aligned}$$

12 $$\begin{aligned} q&= \frac{1}{2}(\Omega +3\left( (\gamma -1)\Omega -\frac{\Omega _A^{\alpha +1}}{\Omega ^\alpha })-2\Omega _\Lambda \right) \end{aligned}$$

13 $$\begin{aligned}&K+\Omega +\Omega _\Lambda = 1 \end{aligned}$$

Here $'$ denotes the derivative with respect to $\tau$.

If $k=0,+1$, the dominant term becomes $D=\sqrt{H^2+{}^3R}$. Thus, for this case we use D, rather than H, to define dimensionless variables as following where *Q* is the dimensionless expansion parameter.

14 $$\begin{aligned} \begin{aligned} Q = \frac{H}{D}, \qquad \Omega =\frac{\mu }{3D^2}, \qquad \Omega _\Lambda =\frac{\Lambda }{3D^2}, \qquad K=-\frac{{}^3R}{6D^2}, \qquad \Omega _A = \dfrac{\root \alpha + 1 \of {A}}{3D^2}, \qquad P=\frac{p}{3D^2} \equiv (\gamma -1)\Omega - \dfrac{\Omega _A^{\alpha +1}}{\Omega ^\alpha } \end{aligned} \end{aligned}$$

If we introduce a time variable as $d\tau =D(t)dt$, we obtain following evolution and constraint equations where $'$ denotes the derivative with respect to $\tau$.

15 $$\begin{aligned} D'&=-\dfrac{3}{2}\left( \gamma \Omega -\dfrac{\Omega _A^{\alpha +1}}{\Omega ^\alpha }\right) QD \end{aligned}$$

16 $$\begin{aligned} Q'&=(Q^2-1)\left[ \dfrac{3}{2}\left( \gamma \Omega -\dfrac{\Omega _A^{\alpha +1}}{\Omega ^\alpha }\right) -1\right] \end{aligned}$$

17 $$\begin{aligned} \Omega '&= 3Q(\Omega -1)\left( \gamma \Omega -\dfrac{\Omega _A^{\alpha +1}}{\Omega ^\alpha }\right) \end{aligned}$$

18 $$\begin{aligned} \Omega '_A&= 3Q\Omega _A\left( \gamma \Omega -\dfrac{\Omega _A^{\alpha +1}}{\Omega ^\alpha }\right) \end{aligned}$$

19 $$\begin{aligned}&\Omega +\Omega _\Lambda = 1 \end{aligned}$$

20 $$\begin{aligned}&Q^2-K = 1 \end{aligned}$$

## Results

For $k=0,-1$ case, since $H'$ equation decouples, we obtain a three-dimensional reduced dynamical system for $\Omega$, $\Omega _\Lambda$ and $\Omega _A$ which are given in Eqs. (9–11). Considering the weak energy condition ($\Omega + P \ge 0$) and $\Lambda \ge 0$ inequality as well as constraint equation 13, we get the following conditions.

21 $$\begin{aligned} \begin{aligned} 0 \le \Omega , \qquad 0 \le \Omega _\Lambda , \qquad 0 \le \Omega + \Omega _\Lambda \le 1, \qquad \Omega _A \le \root \alpha + 1 \of {\gamma }\Omega \end{aligned} \end{aligned}$$

Thus, the state space is three-dimensional and compact. Jacobian matrix becomes as following for this case:

22 $$\begin{aligned} \begin{bmatrix} (6\gamma -4)\Omega -2\Omega _\Lambda -3\gamma -\frac{3\Omega _A^{\alpha +1}(\Omega +\alpha -\Omega \alpha )}{\Omega ^{\alpha +1}}+2 & -2\Omega & -\frac{3\Omega _A^{\alpha }(\Omega -1)(\alpha +1)}{\Omega ^\alpha } \\ (3\gamma -2)\Omega _\Lambda +\frac{3\Omega _\Lambda \Omega _A^{\alpha +1}\alpha }{\Omega ^{\alpha +1}} & (3\gamma -2)\Omega -4\Omega _\Lambda -\frac{3\Omega _A^{\alpha +1}}{\Omega ^\alpha }+2 & -\frac{3\Omega _\Lambda \Omega _A^\alpha (\alpha +1)}{\Omega ^\alpha } \\ (3\gamma -2)\Omega _A+\frac{3\Omega _A^{\alpha +2}\alpha }{\Omega ^{\alpha +1}}& -2\Omega _A & (3\gamma -2)\Omega -2\Omega _\Lambda -\frac{3\Omega _A^{\alpha +1}(2+\alpha )}{\Omega ^\alpha }+2\\ \end{bmatrix} \end{aligned}$$

For $k= 0,+1$ case, $D'$ equation decouples and we get a three-dimensional reduced dynamical system for *Q*, $\Omega$ and $\Omega _A$ which are given in Eqs. (16–18). Considering the weak energy condition ($\Omega + P \ge 0$) and $\Lambda \ge 0$ inequality as well as constraint equation 20, we get the following conditions.:

23 $$\begin{aligned} \begin{aligned} 0 \le \Omega \le 1, \qquad -1 \le Q \le 1, \qquad \Omega _A \le \root \alpha + 1 \of {\gamma }\Omega \end{aligned} \end{aligned}$$

This state space is three-dimensional and compact as well. Jacobian matrix becomes as following for this case:

24 $$\begin{aligned} \begin{bmatrix} -Q(\dfrac{3\Omega _A^{\alpha +1}}{\Omega ^\alpha }-3\Omega \gamma +2) & (Q^2-1)(\frac{3\gamma }{2}+\frac{3\Omega _A^{\alpha +1}\alpha }{2\Omega ^{\alpha +1}}) & -\frac{3\Omega _A^\alpha (Q^2-1)(\alpha +1)}{2\Omega ^\alpha } \\ 3\Omega \gamma (\Omega -1)-\frac{3\Omega _A^{\alpha +1}(\Omega -1)}{\Omega ^\alpha } & 3Q\gamma (2\Omega -1)-\frac{3Q\Omega _A^{\alpha +1}(\Omega +\alpha -\Omega \alpha )}{\Omega ^{\alpha +1}} & -\frac{3Q\Omega _A^\alpha (\Omega -1)(\alpha +1)}{\Omega ^\alpha }\\ 3\Omega \Omega _A \gamma -\frac{3\Omega _A^{\alpha +2}}{\Omega ^\alpha } & 3Q\Omega _A(\gamma +\frac{\Omega _A^{\alpha +1}\alpha }{\Omega ^{\alpha +1}}) & 3Q(\gamma \Omega -\frac{(2+\alpha )\Omega _A^{\alpha +1}}{\Omega ^\alpha }) \end{bmatrix} \end{aligned}$$

These systems of equations seem singular for vacuum solutions but it can be seen that $\Omega =0$ only when $A=0$. In this case where Chaplygin gas is absent, equation of state becomes as in Eq. 25

25 $$\begin{aligned} p=(\gamma -1)\mu \end{aligned}$$

Hence, systems of equations for $A=0$ submanifold become as in Eqs. (26–30) and Eqs. (31–35) for $k=0,-1$ and $k=0,+1$ cases, respectively. These give the same state spaces that are constructed by Goliath and Ellis for perfect fluid matter source^14^.

26 $$\Omega ' = (2q - (3\gamma -2))\Omega$$

27 $$\begin{aligned}\Omega _\Lambda ' = 2(1+q)\Omega _\Lambda \end{aligned}$$

28 $$\begin{aligned}\Omega _A' = 0 \end{aligned}$$

29 $$\begin{aligned}q = \frac{1}{2}((3\gamma -2)\Omega -2\Omega _\Lambda ) \end{aligned}$$

30 $$\begin{aligned}K+\Omega +\Omega _\Lambda = 1 \end{aligned}$$

31 $$\begin{aligned}Q'=(Q^2-1)\left[ \dfrac{3}{2}\gamma \Omega -1\right] \end{aligned}$$

32 $$\begin{aligned}\Omega '= 3Q(\Omega -1)\gamma \Omega \end{aligned}$$

33 $$\begin{aligned}\Omega '_A = 0 \end{aligned}$$

34 $$\begin{aligned}\Omega +\Omega _\Lambda = 1 \end{aligned}$$

35 $$\begin{aligned}Q^2-K = 1 \end{aligned}$$

**Figure 1:**
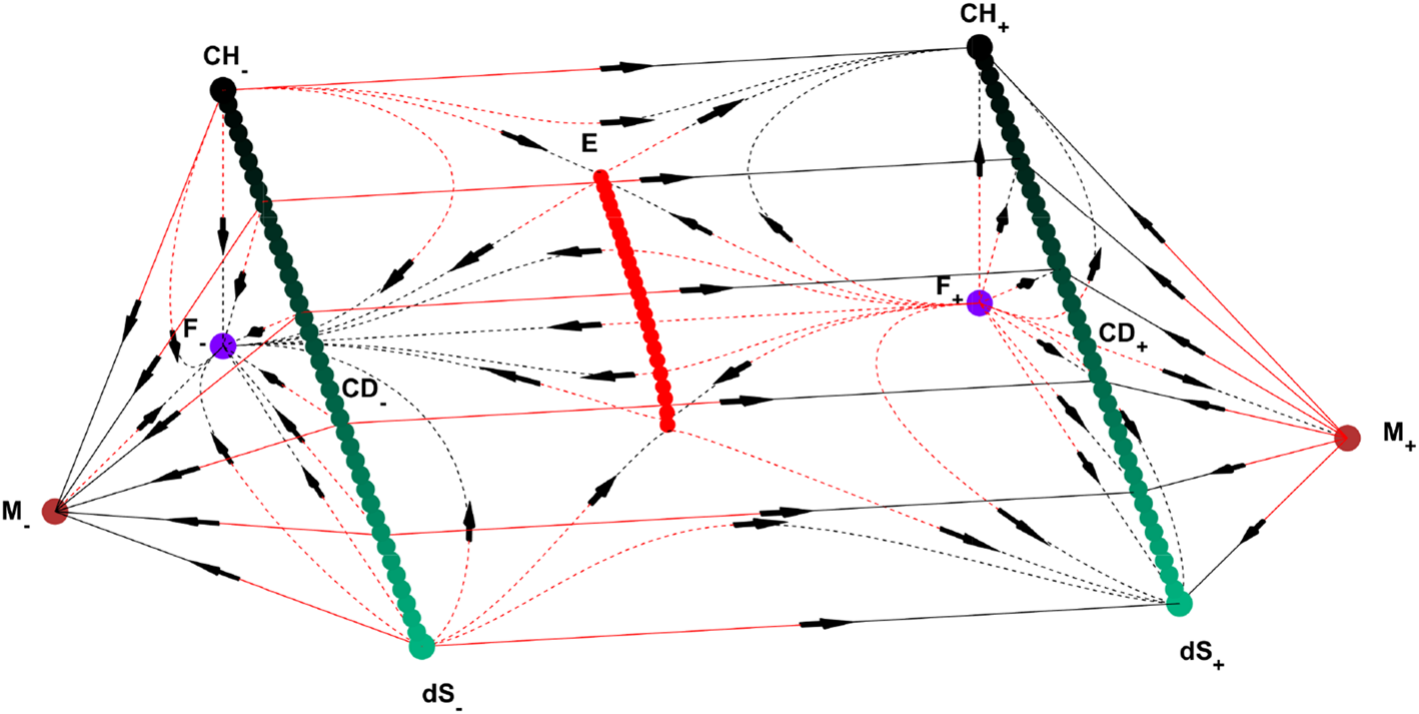
State space for $\alpha =1$ and $\gamma >\frac{2}{3}$. Here, dots denote the solutions of the system; F, M, dS, E, CH and CD represent Flat FL solution$(\Omega =1, \Omega _\Lambda =0, \Omega _A=0, k=0)$, Milne solution$(\Omega =0, \Omega _\Lambda =0, \Omega _A=0, k=-1)$, de Sitter solution$(\Omega =0, \Omega _\Lambda =1, \Omega _A=0, k=0)$, Einstein static solution curve $(H=0, H'=0)$, Chaplygin Gas solution$(\Omega =1,\Omega _\Lambda =0,\Omega _A=\root \alpha + 1 \of {\gamma }, k=0$) and Chaplygin-de Sitter solution line $(\Omega =\Omega ,\Omega _\Lambda =1-\Omega ,\Omega _A=\root \alpha + 1 \of {\gamma }\Omega ,k=0)$, respectively. z axis corresponds to $\Omega _A$ for all of the state space. On $k=+1$ region (middle) vertical and horizontal axes correspond to $\Omega$ and *Q*, respectively. On $k=-1$ region (triangular parts on both sides), M-F and M-dS lines correspond to $\Omega$ and $\Omega _\Lambda$ axes, respectively. The right and left parts of the state space represent expanding and contracting universes, respectively. Subscripts on equilibriums refer to the sign of H there. Lines go from red to black. (MATLAB ver. R2019b).

**Figure 2:**
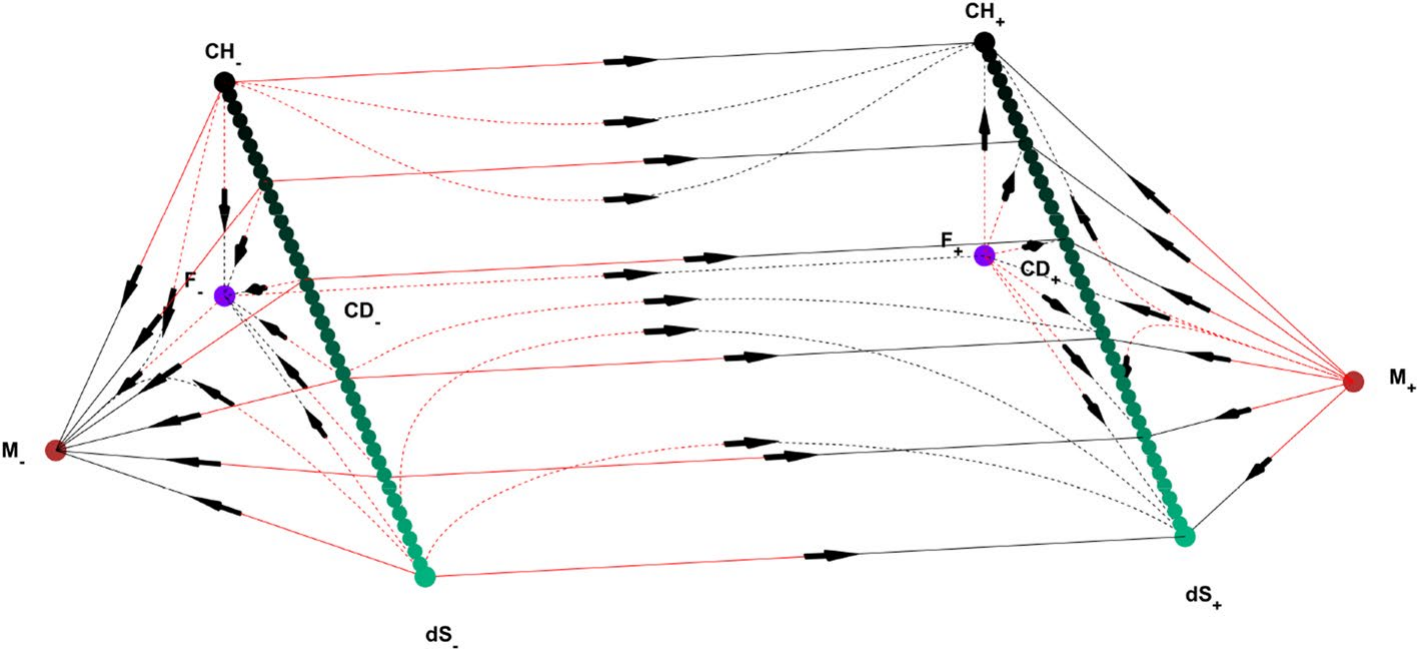
State space for $\alpha =1$ and $\gamma <\frac{2}{3}$. Here, dots denote the solutions of the system; F, M, dS, CH and CD represent Flat FL solution$(\Omega =1, \Omega _\Lambda =0, \Omega _A=0, k=0)$, Milne solution$(\Omega =0, \Omega _\Lambda =0, \Omega _A=0, k=-1)$, de Sitter solution$(\Omega =0, \Omega _\Lambda =1, \Omega _A=0, k=0)$, Chaplygin Gas solution$(\Omega =1,\Omega _\Lambda =0,\Omega _A=\root \alpha + 1 \of {\gamma }, k=0$) and Chaplygin-de Sitter solution line $(\Omega =\Omega ,\Omega _\Lambda =1-\Omega ,\Omega _A=\root \alpha + 1 \of {\gamma }\Omega ,k=0)$, respectively. z axis corresponds to $\Omega _A$ for all of the state space. On $k=+1$ region (middle) vertical and horizontal axes correspond to $\Omega$ and *Q*, respectively. On $k=-1$ region (triangular parts on both sides), M-F and M-dS lines correspond to $\Omega$ and $\Omega _\Lambda$ axes, respectively. The right and left parts of the state space represent expanding and contracting universes, respectively. Subscripts on equilibriums refer to the sign of H there. Lines go from red to black. (MATLAB ver. R2019b).

State space for $k=0, +1$ includes both contracting and expanding cases while state space for $k=0,-1$ depends on the sign of H. Combining one contracting and one expanding state spaces of $k=0,-1$ case with the state space of $k=0,+1$ case on $k=0$ submanifold, we obtain the full dynamical system which is three dimensional and seen in Figs. 1 and 2.

z axis corresponds to $\Omega _A$ for all of the state space. On $k=0,+1$ region (middle) vertical and horizontal axes correspond to $\Omega$ and *Q*, respectively. On $k=0,-1$ region (triangular parts on both sides), M-F and M-dS lines correspond to $\Omega$ and $\Omega _\Lambda$ axes, respectively. The limits of the state space determined by Eqs. (21 and 23) for $k=0,-1$ and $k=0,+1$ cases, respectively.

As seen in Figs. 1 and 2, the dynamical system is bounded by $\Omega _A=0$ (bottom), $\Omega _\Lambda =0$ (back) and $\Omega _A=\root \alpha + 1 \of {\gamma }\Omega$ (front) invariant submanifolds. $k=0$ invariant submanifold lies between the intersection of $k=0,+1$ and $k=0,-1$ state spaces. The state space has a different structure depending on the value of $\gamma$. Since the value of $\alpha$ only changes the positions of Einstein static solutions but does not change the behaviour of the system, we only give $\alpha =1$ case.

The dynamical system has a number of solutions which are given in Tables 1 and 2 where the eigenvalues are determined by the jacobian matrices (22) and (24). Except from E(Einstein static solution curve) which exists for $\gamma >\frac{2}{3}$ and M(Milne solution), all solutions are on the flat submanifold. The pressureless solutions which we call CD(Chaplygin-de Sitter solution) line are at the intersection of flat submanifold and $\Omega _A=\root \alpha + 1 \of {\gamma }\Omega$ pressureless submanifold. CH(Chaplygin gas solution) where $\Omega$ and $\Omega _A$ have their maximum values as well as dS(de Sitter solution) where $\Omega _\Lambda$ has the maximum value, lie at the boundaries of this solution line. The cosmological implications of these equilibriums are given in the Table 3.

**Table 1:** Properties of the equilibrium points for k = 0, $-1$.

	$(\Omega , \Omega _\Lambda ,\Omega _A)$		Eigenvalues			Stability	
			$\lambda _1$	$\lambda _2$	$\lambda _3$	$\gamma$ > 2/3	$\gamma < 2/3$
M	(0, 0, 0)	$H>0$	$2-3\gamma$	2	2	Saddle	Source
		$H<0$	$3\gamma -2$	$-2$	$-2$	Saddle	Sink
F	(1, 0, 0)	$H>0$	$3\gamma -2$	$3\gamma$	$3\gamma$	Source	Saddle
		$H<0$	$2-3\gamma$	$-3\gamma$	$-3\gamma$	Sink	Saddle
CD	$(\Omega ,1-\Omega ,\root \alpha + 1 \of {\gamma }\Omega )$	$H>0$	$-2$	0	$-3\gamma (\alpha +1)$	–	–
		$H<0$	2	0	$3\gamma (\alpha +1)$	–	–

**Table 2:** Properties of the equilibrium points for k = 0,+1. Here, $\xi =(\gamma \Omega ^{\alpha +1}-\frac{2}{3}\Omega ^\alpha )^{\frac{1}{\alpha +1}}$ and $\Sigma =\sqrt{(3\gamma +3\alpha \gamma -2\alpha /\Omega +1)}$ .

	$(Q, \Omega ,\Omega _A)$		Eigenvalues			Stability	
			$\lambda _1$	$\lambda _2$	$\lambda _3$	$\gamma>$ 2/3	$\gamma<$ 2/3
E	$(0,\Omega ,\xi )$	$H>0$	0	$-\Sigma$	$\Sigma$	–	–
	$(0,\Omega ,\xi )$	$H<0$	0	$\Sigma$	$-\Sigma$	–	–
F	(1, 1, 0)	$H>0$	$3\gamma -2$	$3\gamma$	$3\gamma$	Source	Saddle
	$(-1,1,0)$	$H<0$	$2-3\gamma$	$-3\gamma$	$-3\gamma$	Sink	Saddle
CD	$(1,\Omega ,\root \alpha + 1 \of {\gamma }\Omega )$	$H>0$	$-2$	0	$-3\gamma (\alpha +1)$	–	–
	$(-1,\Omega ,\root \alpha + 1 \of {\gamma }\Omega )$	$H<0$	2	0	$3\gamma (\alpha +1)$	–	–

**Table 3:** Cosmological implications of the equilibriums.

F	Flat Friedmann solution	Matter dominated flat universe
dS	de Sitter Solution	$\Lambda$ dominated flat universe
M	Milne solution	The universe with maximum negative curvature
E	Einstein static solution	Static universe with maximum positive curvature
CH	Chaplygin gas solution	Chaplygin gas and matter dominated flat universe
CD	Chaplygin-de Sitter solution line	Pressureless flat universes

**Figure 3:**
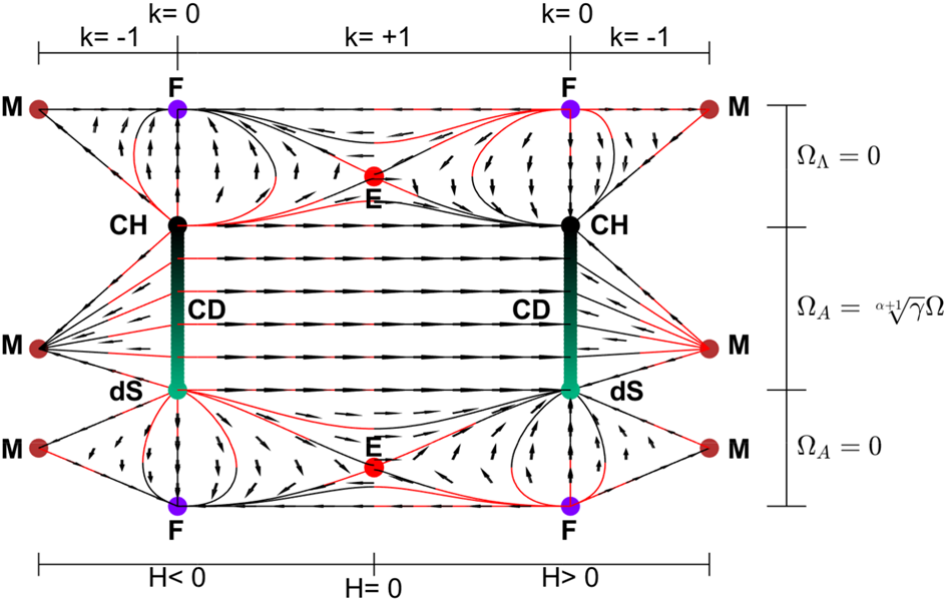
Submanifolds at the boundaries of the state space for $\alpha =1$ and $\gamma >\frac{2}{3}$. Here, dots denote the solutions of the system; F, M, dS, E, CH and CD represent Flat FL solution$(\Omega =1, \Omega _\Lambda =0, \Omega _A=0, k=0)$, Milne solution$(\Omega =0, \Omega _\Lambda =0, \Omega _A=0, k=-1)$, de Sitter solution$(\Omega =0, \Omega _\Lambda =1, \Omega _A=0, k=0)$, Einstein static solution $(H=0, H'=0)$, Chaplygin Gas solution$(\Omega =1,\Omega _\Lambda =0,\Omega _A=\root \alpha + 1 \of {\gamma }, k=0$) and Chaplygin-de Sitter solution line $(\Omega =\Omega ,\Omega _\Lambda =1-\Omega ,\Omega _A=\root \alpha + 1 \of {\gamma }\Omega ,k=0)$, respectively. Lines go from red to black. (MATLAB ver. R2019b).

The state space for $\gamma >\frac{2}{3}$ is seen in Figs. 1, 3 and 5. For $k=0,-1$ and $H>0$, future attractor is the CD equilibrium line. The universes, where $\Omega _\Lambda$ and $\Omega _A$ are both equal to zero, first evolve to Milne model from F(flat Friedmann solution) before expanding to a CD universe. The other universes on this part of the state space expand from F and evolve directly to a CD model. The time reverse region occurs for $H<0$.

For $k=1$ and $H>0$, models which are past asymptotic to F and start with sufficiently small $(\Omega _\Lambda + \Omega _A)$ begin contracting after some expansion and collapse to a big crunch at F while the future attractor for the models with large enough $(\Omega _\Lambda + \Omega _A)$ is a CD universe. For $H<0$, models that start with large enough $(\Omega _\Lambda + \Omega _A)$ cross to $H>0$ region and expand back to their initial conditions while universes with sufficiently small $(\Omega _\Lambda + \Omega _A)$ are future asymptotic to F. There are universes which are past asymptotic as well as future asymptotic to Einstein static model as well.

**Figure 4:**
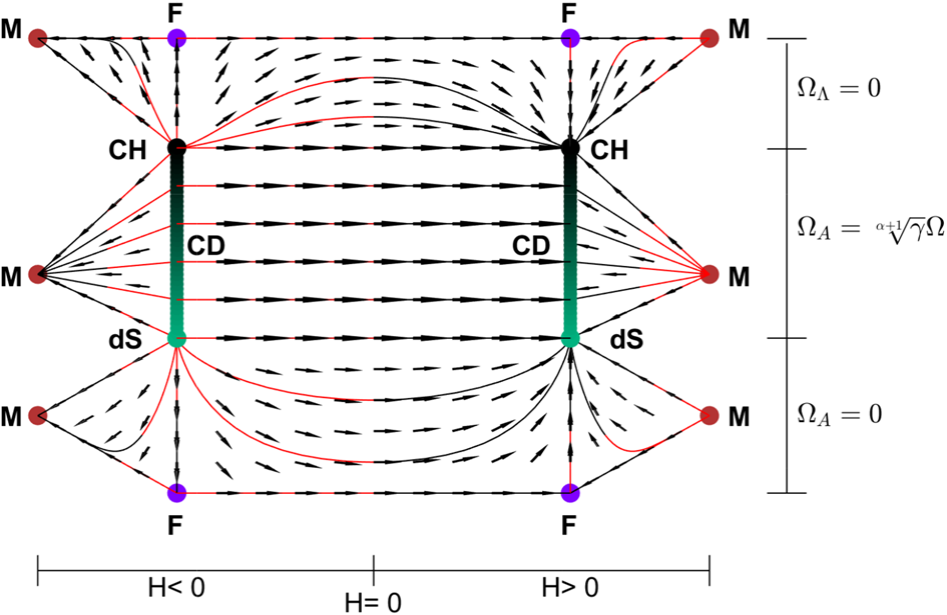
Submanifolds at the boundaries of the state space for $\alpha =1$ and $\gamma <\frac{2}{3}$. Here, dots denote the solutions of the system; F, M, dS, CH and CD represent Flat FL solution$(\Omega =1, \Omega _\Lambda =0, \Omega _A=0, k=0)$, Milne solution$(\Omega =0, \Omega _\Lambda =0, \Omega _A=0, k=-1)$, de Sitter solution$(\Omega =0, \Omega _\Lambda =1, \Omega _A=0, k=0)$, Chaplygin Gas solution$(\Omega =1,\Omega _\Lambda =0,\Omega _A=\root \alpha + 1 \of {\gamma }, k=0$) and Chaplygin-de Sitter solution line $(\Omega =\Omega ,\Omega _\Lambda =1-\Omega ,\Omega _A=\root \alpha + 1 \of {\gamma }\Omega ,k=0)$, respectively. Lines go from red to black. (MATLAB ver. R2019b).

**Figure 5:**
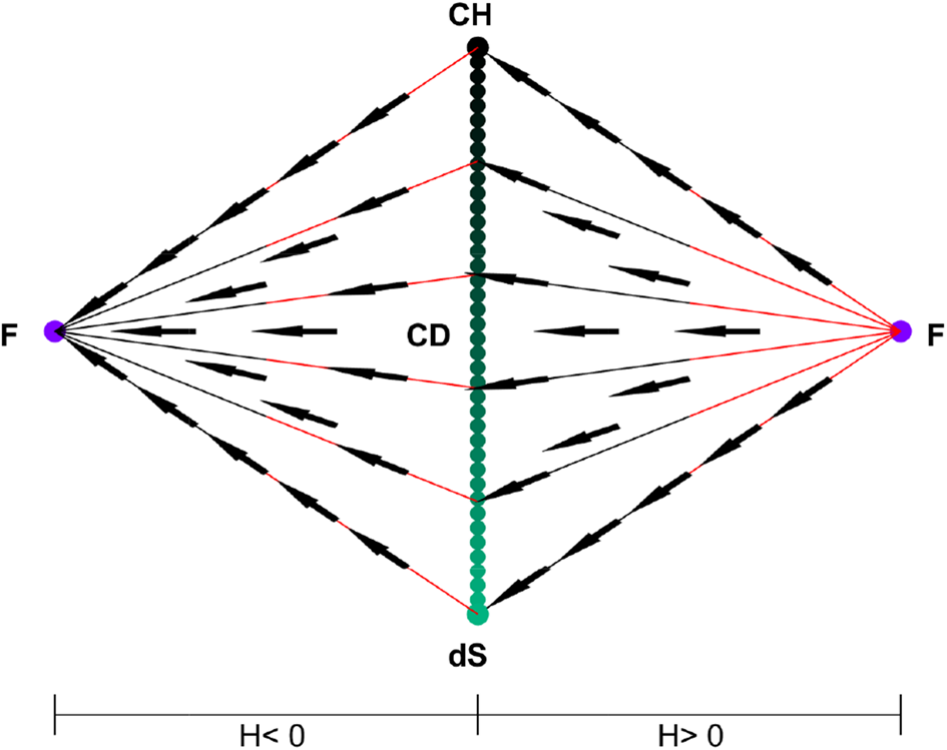
The flat submanifold. Neither $\gamma$ nor $\alpha$ changes the structure of it. Here, dots denote the solutions of the system; F, dS, CH and CD represent Flat FL solution$(\Omega =1, \Omega _\Lambda =0, \Omega _A=0, k=0)$, de Sitter solution$(\Omega =0, \Omega _\Lambda =1, \Omega _A=0, k=0)$, Chaplygin Gas solution$(\Omega =1,\Omega _\Lambda =0,\Omega _A=\root \alpha + 1 \of {\gamma }, k=0$) and Chaplygin-de Sitter solution line $(\Omega =\Omega ,\Omega _\Lambda =1-\Omega ,\Omega _A=\root \alpha + 1 \of {\gamma }\Omega ,k=0)$, respectively. Lines go from red to black. (MATLAB ver. R2019b).

As seen in Figs. 2, 4 and 5 when $\gamma <\frac{2}{3}$,

the structure changes. Einstein static solutions disappear and stability of Milne and flat Friedmann equilibriums change.

For $k=0,-1$ and $H>0$, Milne universe is the source and the universes that are past asymptotic to it, first expand to F and then evolve to a CD universe, if $\Omega _A$ and $\Omega _\Lambda$ are both zero. Otherwise, they expand directly to a CD model. When $H<0$, time reverse region occurs.

For $k=+1$, universes are past asymptotic to contracting versions of the equilibriums as well as future asymptotic to the expanding versions.

## Discussion

For $\gamma >\frac{2}{3}$ case, past attractor for contracting universes is pressureless flat models (CD solution line). From this solution line, if contraction starts with enough$(\Omega _\Lambda +\Omega _A)$ and positive curvature, universe evolve to an expanding CD model. Otherwise, it contracts to matter dominated flat model (Flat Friedmann universe) either directly or first evolving to Milne or Einstein static universes. From this matter dominated flat model, it may bounce back and evolve to contracting flat Friedmann model again or to a CD model depending on the initial condition of the disturbance. Thus, if the universe starts with small $(\Omega _\Lambda +\Omega _A)$ and positive curvature as well as the configuration of the universe does not change at the singularity, universe may bounce back and forth between contracting and expanding flat Friedmann universes. Otherwise, it bounces back and forth between pressureless flat universes (CD solution line) either directly or first evolving to other models.

For $\gamma <\frac{2}{3}$ case, universe expand to a pressureless flat model from either flat Friedmann or Milne universes, if it starts with positive Hubble parameter. Since there are no direction on this solution line, universe may evolve to any model between CH and dS models as long as Hubble parameter is positive. If, it bounces back from here and begin contracting, there are three possible directions for it. If it disturbed to positive curvature, contraction slows down and the universe begin expanding without a singularity and evolve back to expanding CD model. If it starts with zero curvature, universe contracts to flat Friedmann model and from this singularity it may evolve to an expanding flat Friedmann universe or bounce back to an expanding CD model or contracts to Milne universe. From Milne singularity, it expands to a CD universe. Thus, we have a cycle starting with as well as finishing at a pressureless flat model. For both cases, we have a dynamical universe and there is no final state for it as long as we don’t put any restriction on H.

We have examined the evolution of FLRW models with modified Chaplygin gas where the equation of state is $p=(\gamma -1)\mu -\dfrac{A}{\mu ^\alpha }$. It is seen that value of $\alpha$ has negligible effect on the structure of the state space as well as no effect on the stability of the equilibriums. For $\gamma <\frac{2}{3}$, there are 2 equilibrium points which are F and M as well as CD equilibrium line where $\Omega _A=\root \alpha + 1 \of {\gamma }\Omega$. When $\gamma >\frac{2}{3}$, there is an additional equilibrium curve which corresponds to Einstein static universe. Unless we put a restriction on the value of H, there is no stable solution and universe may evolve from one equilibrium point to another continuously but if we assume that Hubble constant is positive, it evolves to a pressureless flat model. We are planning to apply this method to the Bianchi models in future papers.
